# Geography of transnational knowledge flows from China: Distance, Pipelines and Hierarchy?

**DOI:** 10.1371/journal.pone.0326503

**Published:** 2025-06-20

**Authors:** Jiaqian Zhang, Yuefang Si, Jianhui Yu

**Affiliations:** 1 Institute of Geographic Sciences and Natural Resources Research, Chinese Academy of Sciences, Beijing, China; 2 College of Resources and Environment, University of Chinese Academy of Sciences, Beijing, China; 3 School of Geographic Sciences, East China Normal University, Shanghai, China; Maynooth University, IRELAND

## Abstract

With the rise of China’s innovation capacity and position in the global innovation system, an increasing number of scholars are paying attention to the knowledge diffusion from developed economies to China. However, there is less research looking into the destinations of transnational knowledge diffusion from China and their influencing factors from the dynamic perspective. This study uses USPTO data for the period 2003–2022 to illustrate the spatial pattern of Chinese transnational knowledge diffusion and estimates the impact of geographical distance, knowledge pipelines, and hierarchy in the global innovation system. We find that the global innovation system has been comparatively stable, but that China has successfully transitioned from the periphery to being a semi-peripheral and then a core country. The knowledge transfer from China occurred firstly to core and semi-peripheral countries, as the reversed knowledge flow, and then to developing countries along the “Belt and Road” initiative with an increasingly important role in “South-South Cooperation”. Regarding its influencing factors, geographical distance is significant across all periods, highlighting that distance remains an indispensable factor in innovation and knowledge flow. Knowledge pipelines and hierarchy in the global innovation system are conditionally influential. Knowledge pipelines were only significantly positive when China was a semi-peripheral country. Compared with the periphery, the knowledge flow from China increasingly tended towards semi-peripheral countries during its catching-up process, but the knowledge could be accepted by the core countries only during the time when China was a semi-peripheral country. Our research unpacks the complexity of pipelines and hierarchy as influencing factors from the dynamic perspective.

## Introduction

Transnational knowledge diffusion denotes the process of knowledge dissemination, sharing, and transfer across different countries. It has experienced three main historical phases, according to the main flow directions. The first phase started in the 1960s, when it flowed mainly within the triangulation, namely the U.S., Western Europe and Japan. In this period, it reflected the knowledge creation and assimilation in the developed countries for the developed countries [1,2]. A new direction of transnational knowledge diffusion then emerged in the 1990s, defined as the second process, that transferred the vital innovation created in the triangulation to developing countries, and to developing Asia in particular [3,4]. Since the 2010s, the third period has seen knowledge diffusion not only from developed economies, but also from developing Asia, which had been the main destination in the second period. The rapid climb of China in particular on the innovation ladder has received growing research attention [5]. According to an increasing number of scholars, China is shifting from imitator to innovator [6–8], and therefore it has become an increasingly important source of transnational knowledge diffusion. Where does the knowledge flow to? Have the main destinations changed? What are the main factors that influence the geographical distribution of the knowledge diffusion? The geographical distribution and influencing factors of transnational knowledge flows from China have become an increasingly important research topic.

Compared to the accelerating changes of transnational knowledge diffusion, most of the existing literature still mainly focuses on the transnational knowledge from developed countries to China and its contribution to China’s rising innovation capability [9,10]. Only limited research has been done to analyze transnational knowledge diffusion from China to other countries and its influencing factors and consequences. There are three main shortcomings in the research. Firstly, the existing research mainly focus on the role of Chinese multinational enterprises (MNEs), although knowledge diffusion can be facilitated by international trade, patent citation and so on [11–13]. Secondly, most research consists of case studies on individual firms, such as Huawei, or industries, such as solar energy, while large-scale, country-level statistical analyses remain scarce [14,15]. Thirdly, most of research does not take the dynamics view, and therefore it overlooks the impact of external environments in the different historical periods as well [16]. As China’s innovation capabilities improve and its position in the global innovation system changes, whether there are spatial differences in its overseas knowledge diffusion remains an area requiring further research. Therefore, this paper aims to analyze China’s shifting position within the evolutional global innovation systems and to examine the spatial distribution and influencing factors of transnational knowledge diffusion at different stages, thereby exploring how changes in the global innovation landscape affect China’s overseas knowledge acquisition. This work not only provides an important reference for research on China’s knowledge flow but also contributes to a deeper understanding of how to promote and influence knowledge flow in the context of globalization, which has important academic value.

The remainder of the article is organized as follows. After outlining the literature review and detailing the data and methodology, the core section of the article delineates China’s historical evolution within the global innovation landscape and examines the geographic distribution of cross-border knowledge flows across distinct periods. Given that statistical descriptions alone cannot adequately capture the influencing factors behind China’s transnational knowledge flows, we subsequently synthesize the findings addressing the primary research questions along with corresponding insights. The final part presents a consolidation of conclusions alongside an interpretive discussion.

## Literature reviews

According to existing research, knowledge flow on the organizational level is mainly influenced by three factors, namely the knowledge gap between knowledge source and recipient, the knowledge pipeline between them and the absorptive capacity of the knowledge recipient. Knowledge transfer will not happen spontaneously unless both the source and recipient of the knowledge flow have the necessary knowledge base and the appropriate gap [17,18], and the recipient has the ability to learn the knowledge [19].

The knowledge diffusion on the country level is more complicated. The importance of the knowledge gap and absorptive capacity have been widely accepted, since they are the potential needs; however, the importance of knowledge pipelines has been challenged by two related concepts: global knowledge system and geographical distance. These authors claim that the global innovation system has a small club effect, meaning that countries only have intense knowledge exchanges with limited countries, and therefore the hierarchy in the global innovation system, rather than the pipelines, has a strong influence on transnational knowledge diffusion. The geographically close countries have a small club effect too. Within the club, countries share interests, while countries outside the club have potential competition. Therefore, this section will examine the existing research on the effects of geographical distance, knowledge pipelines and hierarchy in the global innovation system on transnational knowledge diffusion.

Geographical distance: the consensus that geographical distance hinders knowledge flow has been widely accepted [20–23]. Tacit knowledge is usually regarded as spatially sticky, as it is effectively transferred only through direct face-to-face encounters [24], meaning that the role of geography must be considered carefully due to the potentially huge cost of transferring this kind of knowledge. While the common belief is that codified knowledge should be transferred across geographical distances easily, some scholars also found that codified knowledge flows, which are captured by patent citations, are spatially bounded [21]. Empirical studies further confirm that codified knowledge diffusion, measured through patent citations, exhibits significant geographical distance decay at national and continental levels, though this effect diminishes in intercontinental contexts where institutional and cognitive proximity may override geographical barriers [25].

Knowledge pipelines: firms can tap into external knowledge in distant parts of the world through many channels, such as trade, licensing, and FDI [26]. As Bathelt et al. [27] pointed out, global pipelines of knowledge may provide firms located in outward-looking location with a string of particular advantages not available to outsiders. Knowledge pipelines are the guarantee of knowledge diffusion. Empirical studies have shown that the effectiveness of knowledge pipelines varies according to the characteristics of the technology, with local knowledge pipelines being more important for technologies rooted in the regional environment and transnational knowledge pipelines being more effective for globalized technologies [28].

Hierarchy (within the global innovation system): the global innovation landscape is highly uneven, exhibiting a core-semi-periphery-periphery hierarchical structure, which is typical of the global innovation system. Some scholars argue that global knowledge diffusion exhibits a “club effect,” primarily flowing among core countries [29], making it difficult for semi-peripheral and peripheral countries to acquire knowledge from core nations [30]. They suggest that countries should target knowledge acquisition from nations at similar developmental levels [31]. Based on the analysis of USPTO utility patent citations from countries around the world, Chen and Guan [29] found that international knowledge flows have a clear core‐peripheral structure. Following this reasoning, it seems that the position in the global innovation network also influences knowledge acquisition. Knowledge is systematically diffused, with the flow of knowledge from the headquarters of Swedish multinationals to smaller non-multinational firms on a global scale seeming to show a similar system. Other authors contend that semi-peripheral and peripheral countries should actively establish knowledge channels with core countries to access innovation resources from these more advanced regions [11–13].

As to the research on China, it remains unclear whether distance, pipeline and hierarchy influence the transnational knowledge diffusion from China during China’s innovation catching up process. Therefore, it is important to analyze the valid of the influencing factors in the different historical periods as to unpack the complexity of the influencing factors.

## Data, methods and variables

### Data

Patents are crucial empirical resources for studying technology-related phenomena, providing rich information on technological innovation and serving as a key indicator of innovative activity [32,33]. Patents contain valuable data, including grant date, title, abstract, inventor, applicant and assignee names and addresses, making them a primary data source for innovation network research. Patent citation is an increasingly important indicator for tracing international knowledge flow direction [34,35]. Among the patent databases, the United States Patent and Trademark Office (USPTO) is internationally recognized as authoritative and the most appropriate for studies on innovation at the global level [36]. USPTO requires inventors to include citations of prior art, has a longer history, or maintains better quality control. The citation information is provided by the USPTO data as well. Therefore, this paper utilizes patent citations registered at USPTO to examine the yearly global innovation system and to define the position of each country, the geographical distribution of transnational knowledge diffusion and so on. The patent data is retrieved from PATSTAT, which was specifically developed by the European Patent Office (EPO) for use by government/intergovernmental organizations and academic institutions and is one of the most widely used patent databases for scholars [37]. PATSTAT covers original patent application information from the EPO, USPTO, Japan Patent Office, and World Intellectual Property Organization databases and comprises a set of tables that follow a relational database schema, allowing users to extract the data on the country, industry, and firm level through Structured Query Language (SQL).

To withdraw the data, we constructed SELECT statements to extract the data on patent applications and patent citations at the country level worldwide from patent applications published by USPTO on the PATSTAT database (Spring 2024 version). More specifically, we use the address of the first-named applicant to determine the origin country of a patent that is cited. We obtain a table describing patent citation relationships at the country level, with the data elements including: (1) the year in which the cited patent was granted, (2) the cited country, (3) the citing country, and (4) the number of times cited. For example, a record (year = 2010, cited country = CN, citing country = JP, times cited = 2927).

**Fig 1:**
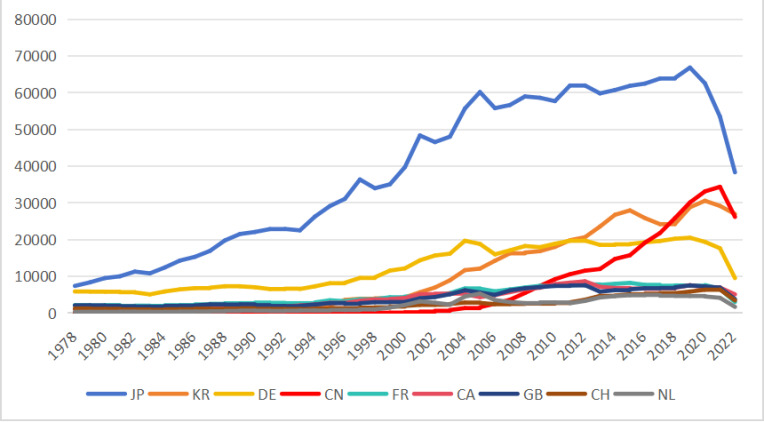
Number of patent applications of some countries in the USPTO from 1978 to 2022.

When analyzing patent data, it is usually necessary to account for the time lag caused by the truncation problem, which typically lasts around three years [38]. As a result, the time span of the study is restricted to the period 2003–2022. Truncation generally refers to the sharp decline in application and citation data caused by the delay in patent applications. It is important to note that patent citations typically occur after a patent is published, but not always. For example, patent A may cite patent B, which is still under review, or patent A may be granted before patent B. These nuances further emphasize the need to address truncation-related time lags in patent analysis [39]. The data from PATSTAT shows that Chinese overseas patent application has been booming in recent years. Prior to 2003, this number was small, but it has grown exponentially since 2003. China successively surpassed Switzerland (2006), the Netherlands (2007), and the United Kingdom (2009); overtook France and Canada (2010); and surpassed Germany and South Korea in 2017–2018. Since then, China has consistently maintained its position as the world’s third-largest patent applicant (shown in Fig 1).

### Methods

This study uses the following two methods. Firstly, we apply the network analysis to examine the yearly global innovation system based on the patent citations data on the country level worldwide from 2003 to 2022, defining the positions of each country, and China in particular. Secondly, we use the fractional regression model to investigate the determinants of Chinese transnational knowledge diffusion.

1) Global innovation system

We draw on Zachary Neal’s method for calculating power and centrality in the network analysis [40]. Using global patent citation data, we classify countries worldwide into three categories (core, semi-peripheral and peripheral countries). Network centrality typically reflects the concentration and distribution of resources, while network power emphasizes the influence and dominance of resource flows. Together, these two concepts help depict the core-periphery structure of the global innovation network.

To avoid the impact of country size, we use the improved Salton index to adjust the patent citation links between countries [41]. The calculation formula is as follows.


$$Cite_{ij}=\frac{C_{ij}}{\sqrt{C_{i}\times C_{j}}}(i,j=1,2,3\ldots ,i\neq j)$$

*Here, Cite*_*ij*_ *represents the patent citation link between countries, C*_*ij*_ *denotes the number of patent citations between country i and country j, which includes the number of patents cited by country i from country j and the number of patents cited by country j from country i. C*_*i*_ *and C*_*j*_ *represent the total number of cross-border patent citations for countries i and j respectively.*

We then construct an inter-country knowledge flow network by treating countries as nodes and the patent citation links between countries as weights. Based on the link weights between countries and the centrality indicators of network nodes, we calculate the centrality and power of each country in the knowledge flow network across years. The specific calculation formula is as follows.

Country centrality,


$$\mathrm{\backslash [}{Centrality}_{i}=\sum_{j=1}^{n}{Cite}_{ij}*{DC}_{j}(j=1,2,3...,n\wedge i\neq j)\mathrm{\backslash ]}$$

Country power,


$$\mathrm{\backslash [}{Power}_{i}=\frac{\sum_{j=1}^{n}{Cite}_{ij}}{{DC}_{j}}(j=1,2,3...,n\wedge i\neq j)\mathrm{\backslash ]}$$

*Here,* ${Centrality}_{i}$ *and* ${Power}_{i}$ *represent the centrality and power of the country i in the knowledge flow network.* ${Cite}_{ij}$ *refers to the patent citation link between country i and country j, while* ${DC}_{j}$ *represents the centrality of country j in the citation network constructed through patent references.*

Finally, we use a system clustering algorithm to classify centrality and power [42]. Countries with strong network centrality and strong power are classified as core countries. Countries with strong centrality but weak power, as well as countries with weak centrality but strong power, are classified as semi-peripheral countries. Countries with weak centrality and weak power are classified as peripheral countries.

2) Variables and regression model

Considering the differences in population scale, economy, technology, patents and innovation direction of different countries, we use the proportion of Chinese patents cited by host countries as the dependent variable (*CnCited*). Considering that the dependent variable is a proportion, this study employs the fractional regression model as the core econometric mode [43], using the commonly adopted “Logistic function” as the link function for calculations and the “Probit function” for robustness tests. Considering the lag effect of innovation and patent citations, all variables in the model are lagged by one year. The econometric model is as follows:


$$CnCited=\alpha +\beta_{1}Cnciting+\beta_{2}Distance+\beta_{3}hierarchy+X+\varepsilon$$ (1)

The dependent variable (*CnCited*) in our model is the proportion of Chinese patents cited in the country. The calculation formula for CnCited is as follows.


$$CnCited_{it}=\frac{C_{it}}{\sum C_{ij}}(i\neq j,j\neq t)$$

*Here, the Cit represents the number of times country i cites Chinese patents, ∑Cij represents the total number of times country i cites patents from other countries, t represents China, and i and j represent countries other than China.*

The three independent variables, namely knowledge pipelines, geographical distance and hierarchy in the global innovation system, that may influence the Chinese transnational knowledge flow, are defined as follows.

Knowledge pipelines: a number of patents cited by China (*Cnciting*) are used as proxies for the knowledge pipelines considering that once China has cited a patent from another country, the two sides have established a knowledge link, thereby “establishing” a knowledge pipeline. The data is extracted from the PATSTAT dataset.

Geographical distance: this is measured by the straight-line distance between the geographic centers of China and the host country (*Distance*). The data is mainly calculated using Google Maps.

Hierarchy in the global innovation system: this is measured by classifying countries according to hierarchical clustering based on the global patent citation data. We divide the hierarchy into three categories, namely core, semi-core and peripheral countries.

In addition, we also include six control variables. Research by some scholars shows that inward FDI or outward FDI, as well as trade, are conducive for promoting cross-border knowledge flow [44–47]. Therefore, total FDI (including inward and outward) between China and other countries (*TFDI*) and total import and export volume of customs goods between China and other countries (*Trade*) are used for controlling knowledge flow in these channels. Technology gap is evaluated by the absolute value of the difference in patent applications between China and the host country (*Technical gap*). Absorptive capacity is depicted by the number of patent applications per capita in the host country (*Patent per capita*) and the proportion of high-tech products exported by the host country (*Tech*). Moreover, we define a technological proximity variable to control mutual citations within the same category and the potential impact of different technological combinations on the model results and to account for the scale, categories, and similarities of innovation and patents between countries. We employ Jaffe’s measurement method to calculate technological proximity between countries [48]. The calculation formula is as follows:


$$S_{it}=\frac{\sum_{k=1}^{m}F_{ki}F_{kt}}{\sqrt{\sum_{k=1}^{m}F_{ki}^{2}\sum_{k=1}^{m}F_{kt}^{2}}}$$

*Here, the t represents time, and i denotes other countries excluding China. S*_*it*_ *indicates the technological proximity between country i and China. The k refers to patent categories, while m represents the number of patent categories. According to the International Patent Classification system (2023 edition) from the National Bureau of Statistics and the classification standards in the PATSTAT database, the data is categorized into 8 sections (m = 8) from Section A to H based on IPC classification codes. F*_*ki*_ *and F*_*kt*_ *represent the number of patent applications in category k for country i and China respectively. The technological proximity index ranges between 0 and 1, with higher values indicating greater technological proximity.*

The definition and data sources of all variables are listed in Table 1.

**Table 1:** Source of variable data and its descriptive statistics.

Variable name	Definition	Source	Mean	Standard Deviation
**Dependent variable**				
CnCited	Proportion of Chinese patents cited in the country	PATSTAT	0.00308	0.00994
**Independent variables**				
Cnciting	Number of patents cited by China from the other country	PATSTAT	678.2	5654.1
Distance	Straight-line distance between the geographic centers of China and the host country	Google Maps	8857.84	3970.02
Hierarchy	The status of the host country in the global innovation system	PATSTAT	1.162	0.474
**Control variables**				
TFDI	Total foreign direct investment between China and the country	World Bank	52196.2	500206.5
Trade	Total import and export volume of customs goods between China and the country	National Bureau of Statistics of China	1895234	5633037
Technical gap	Absolute value of the difference in patent applications between China and the host country	PATSTAT	15041.71	15395.18
Patent per capita	Number of patent applications per capita in the host country	PATSTAT	0.00015	0.00122
Tech	Proportion of high-tech products exported by the host country	World Bank	11.166	24.986
Technical proximity	Technical proximity of the host country to China	PATSTAT	0.29198	0.39315

**Note: The correlation matrix of the variables can be found in the** S1 Appendix.

## Catching-up process of China in the Global innovation system

Table 2 shows the composition of core countries and semi-peripheral countries from 2003 to 2022, as well as the category to which China belongs. We found that over the past two decades, core countries have remained relatively stable, while semi-peripheral countries have experienced frequent changes. The core countries mainly include a small number of developed nations such as the United States, Japan, Germany, France, the United Kingdom, Canada, and South Korea. These countries have maintained a stable core position over the past 20 years, indicating their strong and stable long-term advantages in the global innovation field. In contrast, there are more semi-peripheral countries, and there is a significant dynamic adjustment across different years. Some countries are classified as semi-peripheral in certain years, while in other years, they rise to core countries or fall to peripheral countries. For example, Sweden was initially considered a semi-peripheral country but rose to a core country in 2011, only to fall back to a semi-peripheral country in 2013. Norway, on the other hand, became a semi-peripheral country in 2012 but dropped to a peripheral country in 2018. This suggests that semi-peripheral countries may have stronger volatility.

**Table 2:** Evolution of Core Countries and Semi-peripheral Countries from 2003 to 2022.

Year	Core Country	Semi-peripheral Country	China Category
2003	United States, Japan, Germany, France, United Kingdom, Canada	Republic of Korea, Sweden, Switzerland, Italy, Netherlands, Finland, Israel, Australia, Denmark, Belgium	Peripheral country
2004	United States, Japan, Germany, France, United Kingdom, Canada, Republic of Korea	Sweden, Netherlands, Finland, Switzerland, Italy, Israel, Denmark, Belgium, Singapore, Austria, Australia	Peripheral country
2005	United States, Japan, Germany, France, United Kingdom, Canada, Republic of Korea	Sweden, Netherlands, Finland, Switzerland, Italy, Israel, Denmark, Belgium, Singapore, Austria, Australia	Peripheral country
2006	United States, Japan, Germany, France, United Kingdom, Canada, Republic of Korea	Sweden, Netherlands, Finland, Switzerland, Italy, Israel, Denmark, Belgium, Australia	Peripheral country
2007	United States, Japan, Germany, France, United Kingdom, Canada, Republic of Korea	Sweden, Netherlands, Finland, Switzerland, Italy, Israel, Denmark, Belgium, Singapore, Austria, Australia	Peripheral country
2008	United States, Japan, Germany, France, United Kingdom, Canada, Republic of Korea	Sweden, Netherlands, Finland, Switzerland, Italy, Israel, Denmark, Belgium, Singapore, Austria, Australia, China, India	Semi-peripheral country
2009	United States, Japan, Germany, France, United Kingdom, Canada, Republic of Korea	Sweden, Netherlands, Finland, Switzerland, Italy, Israel, Denmark, Belgium, Singapore, Austria, Australia, China, Bermuda	Semi-peripheral country
2010	United States, Japan, Germany, France, United Kingdom, Canada, Republic of Korea	Sweden, Netherlands, Finland, Switzerland, Italy, Israel, Denmark, Belgium, Singapore, Australia, China, Bermuda, Hong Kong China	Semi-peripheral country
2011	United States, Japan, Germany, France, United Kingdom, Canada, Republic of Korea, Sweden	Netherlands, Finland, Switzerland, Italy, Israel, Denmark, Belgium, Singapore, Australia, China, India, Hong Kong China, Spain	Semi-peripheral country
2012	United States, Japan, Germany, France, United Kingdom, Canada, Republic of Korea, Sweden, Switzerland	Netherlands, Finland, Italy, Israel, Denmark, Belgium, Singapore, Australia, China, India, Hong Kong China, Spain, Austria, Ireland, Norway	Semi-peripheral country
2013	United States, Japan, Germany, France, United Kingdom, Canada, Republic of Korea	Sweden, Netherlands, Finland, Switzerland, Italy, Israel, Denmark, Belgium, Singapore, Austria, Australia, India, Ireland, Norway	Semi-peripheral country
2014	United States, Japan, Germany, France, United Kingdom, Canada, Republic of Korea, Sweden, Switzerland, China	Netherlands, Finland, Italy, Israel, Denmark, Belgium, Singapore, Australia, China, India, Spain, Austria, Ireland, Norway	Semi-peripheral country
2015	United States, Japan, Germany, France, United Kingdom, Canada, Republic of Korea, Switzerland, China	Sweden, Netherlands, Finland, Italy, Israel, Denmark, Belgium, Singapore, Australia, India, Spain, Austria, Ireland, Norway	Core country
2016	United States, Japan, Germany, France, United Kingdom, Canada, Republic of Korea, China	Sweden, Netherlands, Finland, Switzerland, Italy, Israel, Denmark, Belgium, Singapore, Austria, Australia, Hong Kong China, Spain, India, Ireland, Norway	Core country
2017	United States, Japan, Germany, France, United Kingdom, Canada, Republic of Korea, China	Sweden, Netherlands, Finland, Switzerland, Italy, Israel, Denmark, Singapore, Austria, Australia, India, Ireland, Norway	Core country
2018	United States, Japan, Germany, France, United Kingdom, Canada, Republic of Korea, China, Netherlands	Sweden, Finland, Switzerland, Italy, Israel, Denmark, Belgium, Singapore, Austria, Australia, India, Ireland	Core country
2019	United States, Japan, Germany, United Kingdom, Canada, Republic of Korea, China, Netherlands	France, Sweden, Finland, Switzerland, Italy, Israel, Denmark, Belgium, Singapore, Austria, Australia, India, Ireland	Core country
2020	United States, Japan, Germany, France, United Kingdom, Canada, Republic of Korea, China, Netherlands	Sweden, Finland, Switzerland, Italy, Israel, Denmark, Belgium, Singapore, Austria, Australia, India, Ireland, Luxembourg	Core country
2021	United States, Japan, Germany, France, United Kingdom, Canada, Republic of Korea, China, Netherlands	Sweden, Finland, Switzerland, Italy, Israel, Denmark, Belgium, Singapore, Austria, Australia, Hong Kong China, India, Ireland	Core country
2022	United States, Japan, Germany, France, United Kingdom, Canada, Republic of Korea, China	Sweden, Netherlands, Finland, Switzerland, Italy, Israel, Denmark, Belgium, Singapore, Austria, Australia, Hong Kong China, India, Ireland	Core country

China has gradually moved away from its traditional role as a low-cost production base and an innovation peripheral region, achieving a two-step leap from a peripheral country to a core country. Before 2008, China was a peripheral country with a low position in the global innovation system and limited knowledge flow connections with other countries. As China placed increasing emphasis on technological innovation, its innovation status gradually improved, upgrading to a semi-peripheral country with closer knowledge flow connections to other countries (2009–2014). After 2015, China successfully entered the ranks of core countries, with its innovation status becoming increasingly solid, making it an indispensable part of the global innovation system.

Considering the changes in China’s position within the global innovation system, it can be divided into the following three stages: peripheral stage (2003–2008), semi-peripheral stage (2009–2014), and core stage (2015–2022). The geographical distribution and evolution characteristics of China’s transnational knowledge flow in each stage are as follows.

## Transnational knowledge flow from China

**Fig 2:**
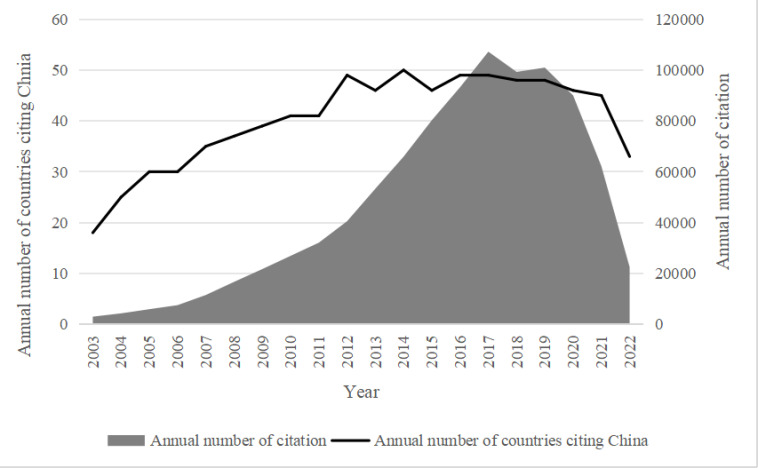
Historical variations of Chinese patent cited countries and citations (2003-2022).

Chinese transnational knowledge has spread widely and has received an increasing amount of attention since 2003. As Fig 2 shows, the number of countries citing Chinese patents shows the overall growth trend, from 17 to a maximum of 50. By 2022, 68 countries have utilized Chinese knowledge. The rising tendency of annual citations and annual cited countries manifests China’s growing capacity in innovation performance and innovation influence.

**Fig 3:**
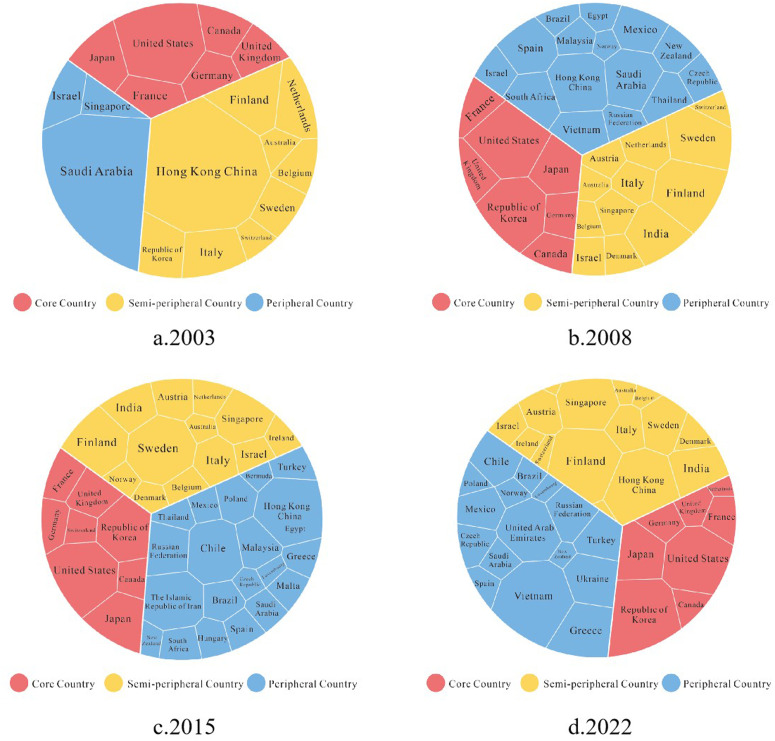
Proportion of Chinese-cited patents by host countries (2003–2021). Note: Subfigure (a) represents the year 2003, (b) the year 2008 (the first year China became a semi-peripheral country), (c) the year 2015 (the first year China became a core country), and (d) the year 2021. The chart is filled with the dependent variable of this study: the ratio of Chinese patent citations to citations of patents from other countries by each host country. Countries are grouped according to their position within the global innovation system in the corresponding year.

During these 20 years, the countries that cited Chinese patents every year were mainly core countries, such as the United States, Japan, Germany, South Korea, and Canada. Simultaneously, the number of forward citations has climbed rapidly and synchronously, increasing around 34 times, from 2965 to 100973(peaking in 2019). Among all the countries citing Chinese patents, the number cited by the US ranks first, up to 607,617 times. Fig 3 presents a pie chart showing the proportion of patents cited by host countries from China, with data for the years 2003, 2008, 2015, and 2021, representatively for each historical periods..

1) Peripheral country (2003–2008)

In this stage, China was at the periphery of the global innovation system, and its transnational knowledge flow was mainly concentrated in a few core and semi-peripheral countries. Among the core countries, the United States held a dominant position, while Japan, Germany, the United Kingdom, France, and Canada were relatively balanced. In the semi-peripheral countries, Hong Kong, Italy, South Korea, and Sweden played a more prominent role. In the peripheral countries, only a few nations, such as Saudi Arabia, exhibited a relatively high proportion. During this period, China’s transnational knowledge flow was primarily concentrated in countries and regions closely linked to its own economy and technology, especially in East Asia, such as Hong Kong, South Korea, and Japan.

2) Semi-peripheral country (2009–2014)

With the rise of China’s innovation status and its entry into the semi-peripheral stage, the geographical distribution of its transnational knowledge flow began to show a trend of diversification. The increase in the number of semi-peripheral and peripheral countries, especially a significant growth in semi-peripheral countries such as Italy, Sweden, and India, reflects China’s gradually strengthening role in global knowledge flow. In this stage, the number of countries involved in China’s transnational knowledge flow nearly doubled, indicating that the spatial scope of its knowledge network was expanding. However, it still primarily relied on core countries in Europe and the United States, along with a few semi-peripheral countries.

3) Core country (2015–2022)

After China became a core country in the global innovation system, the geographical distribution of its transnational knowledge flow exhibited more pronounced characteristics of diversification. Among the core countries, the United States, Japan, and South Korea still show a relatively high proportion. Meanwhile, the number of semi-peripheral and peripheral countries has significantly increased and has remained stable. Notably, countries along the “Belt and Road” initiative have begun to experience greater influence from China’s transnational knowledge flow.

It can be observed that the geographical distribution of China’s transnational knowledge flow has gradually evolved from being highly concentrated to becoming more dispersed and further developed into a phase of diversification and decentralization. This change reflects China’s enhanced position in the global innovation system and the continuous expansion of its knowledge network. At the same time, we find that the role of semi-peripheral and peripheral countries is becoming increasingly important in China’s knowledge flow network, while the dominant influence of core countries is gradually diminishing.

## Fractional regression model estimates of the determinants

### The influencing factors of the Chinese transnational knowledge flow

Table 3 presents the results of the fractional regression model estimation. Models (1–4) present the regression results with control variables added sequentially, while models (5–8) are robust tests using the Probit link function. Although the Probit model indicates that the regression coefficients are underestimated, the overall results of the fractional regression model remain robust.

**Table 3:** Estimation results of the fractional regression model.

Variable	(1)	(2)	(3)	(4)	(5)	(6)	(7)	(8)
*L.Cnciting*	0.237*** (0.019)	0.093*** (0.027)	0.023 (0.024)	0.056** (0.028)	0.092*** (0.007)	0.035*** (0.01)	0.014 (0.009)	0.024** (0.011)
*Distance*	−0.639*** (0.074)	−0.717*** (0.08)	−0.329*** (0.088)	−0.505*** (0.083)	−0.227*** (0.029)	−0.254*** (0.03)	−0.122*** (0.033)	−0.192*** (0.032)
*Hierarchy* *(Semi-peripheral Country)*	1.482*** (0.105)	1.53*** (0.096)	0.682*** (0.107)	0.276*** (0.094)	0.505*** (0.035)	0.528*** (0.031)	0.257*** (0.037)	0.098*** (0.034)
*Core Country*	1.027*** (0.154)	1.042*** (0.17)	0.004 (0.002)	−0.478*** (0.131)	0.361*** (0.049)	0.396*** (0.053)	0.015 (0.049)	−0.163*** (0.047)
*Tech*		0.003*** (0.001)	0.003 (0.002)	0.001 (0.003)		0.001*** (0.001)	0.001* (0.001)	0.0003 (0.001)
*Patent per capita*		0.013*** (0.001)	0.018*** (0.002)	0.018*** (0.002)		0.005*** (0.0003)	0.007*** (0.001)	0.007*** (0.001)
*Technical gap*		0.48*** (0.071)	0.228*** (0.071)	0.3*** (0.072)		0.17*** (0.022)	0.088*** (0.023)	0.111*** (0.024)
*TFDI*			0.05* (0.029)	0.085*** (0.025)			0.017* (0.01)	0.032*** (0.009)
*Trade*			0.369*** (0.055)	0.209*** (0.058)			0.126*** (0.017)	0.07*** (0.019)
*Technical proximity*				1.403*** (0.276)				0.508*** (0.082)
*_Cons*	−0.458 (0.691)	−4.414*** (0.85)	−10.57*** (1.085)	−8.401*** (1.109)	−0.836*** (0.267)	−2.24*** (0.287)	−4.378*** (0.372)	−3.549*** (0.382)
*N*	3135	3135	2338	2338	3135	3135	2338	2338
*Link*	Logit	Logit	Logit	Logit	Probit	Probit	Probit	Probit

Note: The standard error of the estimated coefficient is in parentheses, where ***, ** and * indicate that the estimated coefficient is significant at the level of 1%, 5% and 10% respectively.

Our results show that knowledge pipelines, geographical distance, and the global innovation hierarchy have a significant impact on the Chinese transnational knowledge flow. When other factors are not considered, Model (1) demonstrates the positive effect of knowledge pipelines in promoting knowledge flow, as well as the constraining effect of geographical distance. Meanwhile, the coefficients for semi-peripheral and core countries within the global innovation system (Hierarchy) are both significantly positive. This indicates that China has gained greater recognition from other countries within the global innovation system, gradually transitioning from a peripheral country to a semi-peripheral and even core country, which has significantly facilitated Chinese transnational knowledge flow. In addition, the technological gap between China and the host country is a prerequisite for knowledge flow to occur. When controlling the technological gap, the results of Model (2) remain robust.

Technological proximity is an essential factor that cannot be overlooked when analyzing knowledge flow and the innovation system. This is because innovation and patents often have industry and technological thresholds, and higher technological similarity is more likely to foster technology citation, innovation, and knowledge transfer. Specifically, considering the differences in scale and innovation systems between countries, controlling the “technological proximity” between nations becomes particularly important. Our results also support it. When Model (3) only controls international investment and bilateral trade, significant biases appear in the model’s results, with all core variables except “geographical distance” becoming insignificant. The model’s bias and the instability of coefficients indicate a potential omitted variable bias, suggesting that the model may have overlooked an important variable. Therefore, when we introduce “technological proximity” in Model (4), the results align closely with our prior expectations. Knowledge flow, technological gap, technological proximity, bilateral investment and trade have all significantly facilitated Chinese transnational knowledge flow. However, the hierarchy shows different results. The coefficient for semi-peripheral countries is significantly positive, while the coefficient for core countries is significantly negative. We believe this may reveal the potential competitive and cooperative relationships between China and other countries in innovation and technological patents.

### China’s different catching-up process in the global innovation system

We believe that countries occupying a more central position in the global innovation system often dominate the global innovation landscape. Therefore, integrating into the global innovation system inevitably requires recognition from core countries. China became a semi-peripheral country in 2009 and advanced to a core country in 2015. We aim to explore whether transnational knowledge flow is more active between semi-peripheral and core countries during China’s different catching-up process in the global innovation system in order to analyze whether hierarchy might act as a barrier to innovation activities.

Table 4 shows the model results for China at different levels of hierarchy within the global innovation system. We find that “technological proximity” is the most important factor influencing China’s transnational knowledge flow, which is largely consistent with the conclusions from Table 3. When China was a peripheral country in the global innovation system, the coefficients for semi-peripheral and core countries were both significantly negative (Model-10), with the coefficient for core countries being even smaller. This indicates that the innovation activities of semi-peripheral and core countries did not cite China’s patents more frequently, and the impact of China’s transnational knowledge flow in these countries was very limited. Between 2009 and 2015, China became a semi-peripheral country in the global innovation system. In this period, the coefficient for semi-peripheral countries was positive but not significant, while the coefficient for core countries remained significantly negative (Model-12). It is important to note that compared to Model (10), the coefficient for core countries significantly decreased. After China became a core country in 2015, the coefficient for semi-peripheral countries became significantly positive, while the coefficient for core countries was negative but not significant (Model-14).

**Table 4:** Transnational knowledge flow in different catching-up processes.

Variable	(9)	(10)	(11)	(12)	(13)	(14)
*L.Cnciting*	0.752*** (0.121)	0.268* (0.159)	0.237*** (0.036)	0.12* (0.066)	0.182*** (0.02)	0.037 (0.034)
*Distance*	−0.861*** (0.199)	−0.356* (0.219)	−0.574*** (0.071)	−0.418*** (0.071)	−0.701*** (0.101)	−0.611*** (0.141)
*Hierarchy* *(Semi-peripheral Country)*	−0.314 (0.225)	−0.599*** (0.226)	0.255** (0.103)	0.036 (0.096)	0.663*** (0.136)	0.434*** (0.126)
*Core Country*	−1.133*** (0.328)	−1.66*** (0.335)	−0.308* (0.175)	−0.548*** (0.201)	0.122 (0.172)	−0.12 (0.172)
*Technical proximity*	3.473*** (0.561)	2.263** (0.951)	2.696*** (0.284)	2.568*** (0.397)	1.398*** (0.249)	1.08*** (0.287)
*Tech*		−0.022** (0.01)		0.004 (0.004)		0.0006 (0.004)
*Patent per capita*		0.091*** (0.142)		0.025*** (0.004)		0.017*** (0.002)
*Technical gap*		0.016 (0.142)		0.062 (0.072)		0.201* (0.119)
*TFDI*		0.012 (0.064)		0.029 (0.032)		0.066 (0.046)
*Trade*		0.421* (0.224)		0.104 (0.083)		0.237*** (0.08)
*_Cons*	−1.498 (1.953)	−10.399*** (3.402)	−2.408*** (0.632)	−5.899*** (1.382)	0.175 (0.994)	−6.41*** (1.479)
*China*	P	P	S-P	S-P	Core	Core
*N*	825	533	990	786	1320	1019

Note: The standard error of the estimated coefficient is in parentheses, where ***, ** and * indicate that the estimated coefficient is significant at the level of 1%, 5% and 10% respectively. “S-P” represents the semi-peripheral country, and “P” represents the peripheral country.

Our results confirm that hierarchy is one of the key factors influencing China’s transnational knowledge flow. Models (9–14) also reveal the existence of potential competitive and cooperative relationships between China and semi-peripheral as well as core countries. During China’s catching-up process in the innovation system, it gradually established strong cooperative relationships with semi-peripheral countries, and transnational knowledge flow had a significant impact in these countries. In contrast, China has consistently lacked effective influence over core countries. When China was technologically lagging (as a peripheral country), it was excluded from innovation cooperation by core countries. After China became a semi-peripheral country, this situation improved to some extent, but it was still not a major cooperation partner for core countries. Finally, when China became a core country, it turned into a competitor for other core countries, with competitive relationships significantly outweighing cooperative ones.

### Heterogeneity of semi-peripheral and core countries

To further analyze China’s potential competitive and cooperative relationships within the global innovation system, we conducted subsample regression (Table 5). Models (15−17) and (18−20) represent the regression results for China as a semi-peripheral country and as a core country respectively. Firstly, knowledge pipelines are only significantly positive when China is a semi-peripheral country, with larger coefficients for peripheral and semi-peripheral countries (Models 15−17). After China became a core country, the coefficient of the knowledge pipelines variable showed changes in sign and became insignificant (Models 18−20). Secondly, geographical distance is significant across all periods, highlighting that distance remains an indispensable factor in innovation and knowledge flow. Finally, the results for technological proximity are consistent with our prior expectations. In most cases, higher technological proximity is more conducive to promoting innovation and knowledge flow. However, Model (20) reveals a unique situation. As mentioned in Table 2 and its explanatory text, after China became a core country, its relationship with other core countries shifted from cooperation to competition. This competitive relationship is directly reflected in the coefficient for technological proximity, which is significantly negative (−4.549).

**Table 5:** The results of the subsample regression.

Variable	(15)	(16)	(17)	(18)	(19)	(20)
*L.Cnciting*	15.317** (7.215)	4.339*** (0.967)	0.181** (0.071)	−0.165 (9.792)	3.259*** (0.505)	0.071 (0.055)
*Distance*	−0.439*** (0.134)	−1.11*** (0.078)	−0.461** (0.184)	−0.619** (0.304)	−1.624*** (0.164)	−0.33** (0.156)
*Tech*	0.007* (0.004)	−0.009** (0.004)	0.007 (0.011)	−0.005 (0.009)	0.011*** (0.003)	0.028* (0.015)
*Patent per capita*	0.026*** (0.005)	0.137*** (0.037)	0.211* (0.111)	0.019*** (0.004)	−0.028 (0.034)	−0.04 (0.079)
*Technical gap*	0.807*** (0.31)	0.555*** (0.15)	0.021 (0.05)	0.941* (0.567)	0.984*** (0.158)	0.01 (0.053)
*TFDI*	−0.027 (0.051)	0.029 (0.024)	0.188*** (0.035)	0.121 (0.086)	−0.067*** (0.026)	−0.042 (0.065)
*Trade*	0.18* (0.105)	−0.024 (0.05)	−0.515*** (0.086)	0.246** (0.102)	−0.097 (0.061)	0.255 (0.322)
*Technical proximity*	2.286*** (0.397)	3.476*** (0.604)	0.553 (3.339)	1.256*** (0.293)	0.662*** (0.215)	−4.549*** (0.858)
*_Cons*	−13.153*** (3.235)	−3.557** (1.77)	3.915 (5.643)	−14.51*** (4.429)	1.298 (2.069)	−1.433 (6.596)
*China*	S-P	S-P	S-P	Core	Core	Core
*Hierarchy*	P	S-P	Core	P	S-p	Core
*N*	672	70	44	860	100	59

Note: The standard error of the estimated coefficient is in parentheses, where ***, ** and * indicate that the estimated coefficient is significant at the level of 1%, 5% and 10% respectively. “S-P” represents the semi-peripheral country, and “P” represents the peripheral country.

## Conclusion and discussion

With the improvement of innovation capabilities in emerging countries led by China, some scholars are beginning to call attention to the flow of knowledge from these countries [49,50]. This study constructs a new global innovation system based on the patent citation on the country level worldwide spanning from 2003 to 2022, analyzes the geographical pattern of Chinese transnational knowledge diffusion.We find firstly that the core of the global innovation system has been dominated by Western Europe (Germany, United Kingdom, and Sweden), North America (United States and Canada) and East Asia (China, Taiwan, Japan, and South Korea). China is the only country that has successfully transitioned from the periphery (2003–2008) to being a semi-peripheral country (2009–2014) and then a core country (2015–2022) of the global innovation system.Secondly, transnational knowledge diffusion from China initially flowed mainly to core and semi-peripheral countries, and then diversified into developing countries along the “Belt and Road” initiative as well from 2015, when China became a core country in the global innovation system. It proves that the transnational knowledge diffusion between China and developed countries is not a one-way street, but rather a two-way interaction [9,51,52], with China playing an increasingly important role in “South-South Cooperation”.

Moreover, we contribute the theoretical research on the influencing factors of transnational knowledge diffusion from the dynamic perspective. We prove the importance of geographic proximity and unpack the complexity of knowledge pipelines and hierarchy. Knowledge pipelines were only significantly positive when China was a semi-peripheral country. As for hierarchy in the global innovation system, China gradually established strong cooperative relationships with semi-peripheral countries during its catching-up process in the innovation system, but the knowledge could be accepted by the core countries only during the time when China was a semi-peripheral country. In other words, the knowledge from China is not accepted by core countries, despite good pipelines, due to the potential competition and diverse industrial and technological development strategies. By doing these, we also prove the specialty and complexity of transnational knowledge diffusion from China, that is the interplay between knowledge pipeline and hierarchy. According to the existing research which were mainly based on the empirical evidence into developed countries and core countries, technological pipeline has positive relation with transnational knowledge diffusion. However, the knowledge diffusion from China is influenced by its position in global innovation system as well, which causes the invalid of pipeline under some conditions, due to the potential competitions and different technological development strategies.

These findings can be informative for policy-makers and managers aiming to enhance competitiveness in the global innovation system. When it comes to the promotion of obtaining knowledge, policy-makers should focus on the countries that has good knowledge pipelines, short geographical distance, and should consider the influence of hierarchy in the global innovation system as well. The demand for domestic pioneer innovative capacity is as important as obtaining transnational knowledge. Managers should establish various knowledge strategies, such as establishing knowledge pipeline to enhance the transnational knowledge communication with counterparts in semi-peripheral countries, developing different knowledge strategies with counterparts in core countries, and transferring knowledge to developing countries as much as possible as to enhance the influence Chinese technology and standards.

Although the results of the study are interesting, it has some limitations. Firstly, although the patent data is a good proxy for studying the flow of knowledge, it cannot reflect the knowledge flow in the real world comprehensively because of the presence of tacit knowledge [53]. Citation may occur without knowledge diffusion, when citations are added by patent examiners, who have broader knowledge of existing patents, even without the inventor’s awareness. Empirical study on the data besides patent are need to further test the valid of our findings. Secondly, the data used for analysis is country-level, which can shed light on the macro dynamics of Chinese transnational knowledge flow, but cannot clarify the micro-mechanisms. The patents on the technology family level can be further explored in the further as well.

## Supporting information

S1 File Data statement. All patent-related data in the study are drawn from the published database. For detailed information, please refer to the provided file.
(DOCX)

S1 Appendix Correlation matrix of variables. (DOCX)
