# Supplementary material for: Geography of transnational knowledge flows from China: Distance, Pipelines and Hierarchy?
Source: PLoS One. 2025 Jun 20;20(6):e0326503. doi: 10.1371/journal.pone.0326503 (PMC12180624; doi:10.1371/journal.pone.0326503)
Supplement: S1 File — All patent-related data in the study are drawn from the published database. For detailed information, please refer to the provided file. (DOCX) [file pone.0326503.s001.docx]

**Data Statemen**t

All patent-related data in the study are drown from the published database, including Worldwide Patent Statistical Database (PATSTAT, <https://data.epo.org/expert-services/>), National Bureau of Statistics of China (https://data.stats.gov.cn/), World Bank(<https://data.worldbank.org/>), and Google Maps (<https://www.google.com/maps/>). The detail information please refer to the following tables.

**Supporting Table 1 Source of variable data and its descriptive statistics.**

| **Variable name** | **Definition** | **Source** |
| --- | --- | --- |
| CnCited | Proportion of Chinese patents cited in the country | PATSTAT（https://data.epo.org/expert-services/） |
| Cnciting | Number of patents cited by China from the other country | PATSTAT（https://data.epo.org/expert-services/） |
| Distance | Straight-line distance between the geographic centers of China and the host country | Google Maps (https://www.google.com/maps/) |
| Hierarchy | The status of the host country in the global innovation system | PATSTAT（https://data.epo.org/expert-services/） |
| TFDI | Total foreign direct investment between China and the country | World Bank (https://data.worldbank.org/) |
| Trade | Total import and export volume of customs goods between China and the country | National Bureau of Statistics of China (https://data.stats.gov.cn/) |
| Technical gap | Absolute value of the difference in patent applications between China and the host country | PATSTAT（https://data.epo.org/expert-services/） |
| Patent per capita | Number of patent applications per capita in the host country | PATSTAT（https://data.epo.org/expert-services/） |
| Tech | Proportion of high-tech products exported by the host country | World Bank (https://data.worldbank.org/) |
| Technical proximity | Technical proximity of the host country to China | PATSTAT（https://data.epo.org/expert-services/） |
