# Supplementary material for: Geography of transnational knowledge flows from China: Distance, Pipelines and Hierarchy?
Source: PLoS One. 2025 Jun 20;20(6):e0326503. doi: 10.1371/journal.pone.0326503 (PMC12180624; doi:10.1371/journal.pone.0326503)
Supplement: S1 Appendix — (DOCX) [file pone.0326503.s002.docx]

Appendix

**Appendix Table 1: Correlation Matrix of Variables**.

| Variable name | *CnCited* | *Cnciting* | *Patent per capita* | *Tech* | *Distance* | *Hierarchy* | *TFDI* | *Trade* | *Technical gap* | *Technical proximity* |
| --- | --- | --- | --- | --- | --- | --- | --- | --- | --- | --- |
| *CnCited* | 1 |  |  |  |  |  |  |  |  |  |
| *Cnciting* | 0.2485 | 1 |  |  |  |  |  |  |  |  |
| *Patent per capita* | 0.0981 | 0.0471 | 1 |  |  |  |  |  |  |  |
| *Tech* | 0.1695 | 0.0378 | 0.1412 | 1 |  |  |  |  |  |  |
| *Distance* | -0.1518 | -0.033 | -0.1179 | 0.0575 | 1 |  |  |  |  |  |
| *Hierarchy* | 0.2919 | 0.439 | 0.1173 | 0.0323 | -0.1426 | 1 |  |  |  |  |
| *TFDI* | 0.2494 | 0.0709 | 0.0874 | 0.0522 | -0.1056 | 0.0695 | 1 |  |  |  |
| *Trade* | 0.4342 | 0.7427 | 0.118 | 0.0161 | -0.1822 | 0.5739 | 0.3818 | 1 |  |  |
| *Technical gap* | 0.2274 | 0.6427 | 0.0367 | 0.0478 | 0.0181 | 0.2143 | 0.0738 | 0.5266 | 1 |  |
| *Technical proximity* | 0.2728 | 0.182 | 0.1337 | -0.026 | -0.0458 | 0.5158 | 0.0839 | 0.3469 | 0.0461 | 1 |
